# A framework for community health worker optimisation in conflict settings: prerequisites and possibilities from Northwest Syria

**DOI:** 10.1136/bmjgh-2023-011837

**Published:** 2023-07-05

**Authors:** Ahmad Habboush, Abdulkarim Ekzayez, Brynne Gilmore

**Affiliations:** 1 Research for Health System Strengthening in Syria, UOSSM, Gaziantep, Turkey; 2 Health Systems, Syria Public Health Network, London, UK; 3 War Studies, King's College London, London, UK; 4 UCD Centre for Interdisciplinary Research, Education and Innovation in Health Systems (UCD IRIS), School of Nursing, Midwifery and Health Systems, University College Dublin, Dublin, Ireland

**Keywords:** qualitative study, health education and promotion, health policy, health systems, health services research

## Abstract

**Background:**

The world will face a human resource gap of 10 million health workers in 2030. Community health workers (CHWs) can contribute to mitigating this workforce gap while improving equitable access to care and health outcomes. However, questions on how to best implement and optimise CHW programmes, especially across varied contexts, remain. As each context has its determinants for a successful CHW programme, this research identifies and assesses pertinent factors needed for optimal CHW programmes in conflict settings, specifically Northwest Syria.

**Methods:**

A mixed-methods study in Northwest Syria consisting of a literature and document review, semistructured interviews with CHWs’ team leaders and programme managers, key informant interviews with policymakers and a survey with CHWs was conducted across three research phases from 2018 to 2022. The three phases aimed to identify, refine and finalise a framework for CHW optimisation in humanitarian conflict contexts, respectively. Qualitative data were analysed thematically, and quantitative data were statistically analysed to identify critical trends.

**Results:**

16 interviews and 288 surveys were conducted, supplemented by key reports and literature. The framework underwent two iterative rounds of refinement, reflecting varying stakeholders’ perceptions of CHW optimisation. The resulting framework presents important implementation factors with subthemes across identified topics of institutionalisation, integration and representation for CHW optimisation in Northwest Syria and other humanitarian conflict contexts. The presented factors are similar in various ways to other fragile low/middle-income country settings. However, in protracted conflict settings like Syria, careful consideration should be given to strategic dimensions such as integration and representation.

**Conclusion:**

For CHW programmes to impact health outcomes in humanitarian conflict settings, they require a set of implementation and design factors relevant to the context. The dynamics of humanitarian funding restrictions, health system capacity and governance structures confront achieving these requirements. Nevertheless, pioneering projects which use available resources are possible. Evidence is needed to understand the impact of CHWs’ interventions and further support implementation across humanitarian contexts.

**WHAT IS ALREADY KNOWN ON THIS TOPIC**

- Evidence shows that community health workers (CHWs) have significantly improved public health conditions in numerous low/middle-income countries and high-income countries. However, the optimisation of these programmes remains a key challenge across settings.
- Most of the evidence comes from settings with a governance system. However, there is little evidence of CHW programme optimisation in humanitarian non-government areas, specifically Syria.

**WHAT THIS STUDY ADDS**

- This study presents a framework for optimising CHW programmes in Northwest Syria as a humanitarian setting characterised by protracted conflicts, lack of governance and dependence on international aid for health service delivery. It highlights and explains the need for institutionalisation, integration and representation (impact measurements).

**HOW THIS STUDY MIGHT AFFECT RESEARCH, PRACTICE OR POLICY**

- The study highlights critical challenges confronting the optimal use of CHW programmes in Northwest Syria, including limited funding, weak governing structures and stakeholders’ perception. Accordingly, the produced framework calls for the collaborative implementation of exemplary CHW programmes from which evidence can be generated and used for advocacy.

## Introduction

Current health resources are inadequate to meet the Sustainable Development Goals, including access to universal healthcare. In addition, health workforce shortage projections are 18 million in 2016, 15 million in 2022 and 10 million in 2030.^1^ Consequently, there has been an increased interest in various task-shifting approaches for health service delivery. Community health workers (CHWs) have received increased attention.^2 3^ CHWs are most relevant where health resources are scarce, access is challenged and equity is compromised. Evidence of CHWs as effective interventions in various fields,^4^ such as maternal and child,^5^ HIV and AIDS,^6^ nutrition,^7^ acute diarrhoeal diseases^8^ and non-communicable diseases,^9–13^ is abundant.

### CHWs in humanitarian and conflict contexts

Humanitarian contexts in this paper adapt the definition of the interagency standing committee as a total or considerable breakdown of authority resulting from internal or external conflict and which requires an international response that goes beyond the mandate or capacity of any single agency.^14^

Studies from conflict settings where formal health systems are temporarily weakened or dysfunctional have shown that CHWs’ role is critical in providing community-based services.^15 16^ In addition, CHWs have the potential to play an essential role in the preparedness and response to emergencies, including outbreaks^17^ or natural disasters,^18^ which are usually characterised by an urgent demand to enhance access to essential healthcare services in hard-to-reach areas without basic infrastructures.^19^ Conflict-related conditions such as increasing insecurity, multiple rounds of displacement, disturbed supply chains, and normalised attacks on health facilities and health professionals can all exacerbate pre-existing shortages of human and financial resources.^20^ The politicisation of health is also witnessed in conflict settings where governments usually neglect opposition-controlled areas, like in El Salvador, Ethiopia and Myanmar.^21^ Examples from Yemen and South Sudan presented the potential CHWs (based in their communities) have in supporting the health system and community resilience, continuing essential services and effective emergency response.^15 22^

Even though CHWs can play essential preventive, promotive and curative roles in various fragile contexts, evidence on the specific circumstances and factors impacting CHWs’ functionality in these contexts is limited. Even more so than in non-fragile settings, health system and community support influence CHW performance.^23^ Integration into the broader healthcare service delivery system, including embeddedness and connectivity, is also a critical foundational element of successful CHW programmes.^2^ The WHO 72 Assembly noted with concern ‘the uneven integration of community health workers into health systems, as well the limited use of evidence-informed policies, international labour standards and best practices to inform the education, deployment, retention, management and remuneration of community health workers, and noting the impact this may have on access to services, quality of health services and patient safety’.^9^

### CHWs in Syria

Similar in many ways to CHWs in other conflict settings, CHWs in Syria are a product of the context in which they operate. The Syrian conflict, a prolonged humanitarian crisis, has displaced more than half of the population. The civil movement in 2011 later developed into international armed conflict amid conflicted political agendas.^24^ The economic crisis exacerbated by forced displacement, COVID-19, the cholera outbreak and recently a devastating earthquake (February 2023) has jointly added layers to the already complex life conditions of over 4.5 million persons living in Northwest Syria. More than 2.8 million are internally displaced people, many living in condensed camps with limited access to essential services.^25^ Women and children compose the majority (79%) of this caseload. According to the disability prevalence and impact report using the Washington Group, 3.7 million persons over 12 years have a disability. In the Northwest, it varies from 22% in Idlib to 26% in Aleppo.^26^ Like other conflicts, this war has hardened the non-government-controlled areas’ health systems. Fifty per cent of health facilities were destroyed, and 70% of healthcare providers fled the country.^16 20 27^ Consequently, the workload and mental and physical pressure took a toll on the remaining health professionals.^28^ The said conditions have confronted accessibility, equity, service continuity and utilisation of health services.^25^

Counting on bottom-up solutions to mitigate the mentioned challenges was common in similar crises.^21^ CHWs are one of those solutions presented in the Syrian conflict. However, while CHWs were adopted, governed, and supported directly or in partnership with governments in Afghanistan, South Sudan, or Iraq, they were introduced to Northwest Syria by international organisations with little genuine participation from local actors. In addition, the concept of CHWs was unfamiliar to the community and was not led by an overarching governing entity with executive power. These critical conditions hindered CHWs’ integration into the broader health system. Thus, limited literature is available to describe CHWs in Syria generally or in the Northwest region even before the conflict.

Consequently, researchers were challenged to identify dedicated CHWs’ policies and guidelines for Syria. The essential primary healthcare package developed by the WHO for Northwest Syria in 2017 did refer to CHWs as an auxiliary component linked to primary health centres (PHCs).^29^ However, it did not identify CHWs’ roles and responsibilities or how to integrate them into the broader system. This gap was rectified by the CHW training manual developed in 2018 in coordination between non-governmental organisations (NGOs) and the WHO. The manual described the role of CHWs and linked them to PHCs. However, the issue remained since no official entity monitors the overall implementation or ensures adherence to this manual.

More importantly, there was little to no effort to produce empirical evidence on CHWs’ relevance, impact, performance, motivation and possible ways to optimise their role. However, the importance of such evidence has been increasingly acknowledged in the international community as it is cited as a fundamental aspect of CHW programme optimisation.^30^

To this end, this paper identifies and explores contextually the operational and strategic factors required for CHWs in Northwest Syria as a fragile context characterised by protracted conflicts and a lack of governance structures, filling a critical knowledge gap.

## Methods

**Figure 1:**
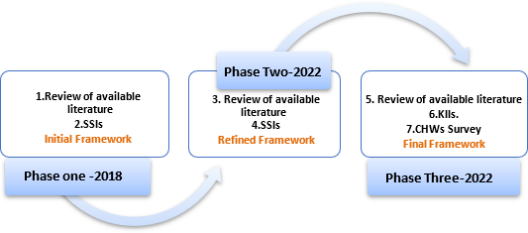
Iterative research approach toward the final framework. SSIs, Semi Structured Interviews; KIIs, Key Informant Interviews; CHWs, Community Health Workers.

A mixed-methods, iterative research approach was used to develop the framework for CHW optimisation. This study occurred in three phases from June 2018 to August 2022 in Northwest Syria and Turkey. The phases were iterative in that the initial phases’ framework contributed to the design and development of the subsequent phases’ data collection and tools. Figure 1 details the overall methodological approaches used to develop the framework. Details on each phase are described below. A qualitative interpretive approach was used to understand individuals’ perceptions and ideas of the world around them.^31^ Phase one findings were supplemented in phases two and three using a mix of surveys, semistructured interviews (SSIs), key informant interviews (KIIs) and a review of available literature. SSIs were selected because they enabled the researcher to gather open-ended data, explore participant thoughts, feelings and beliefs about the topic, and deeply explore personal issues.^32^ Conducting these interviews with key informants in the last phase was critical as that phase’s purpose was to critique previous phases’ findings and include the perspectives of expert policy and decision-makers from the Northwest. In addition, it was necessary to present findings from the field to those in the decision-maker positions, which helped raise their awareness and enthusiasm about the topic.^33 34^

For the interviews in all three phases, initial approval for participation was gained by phone and followed up with email communication to formalise the participation. Interviews were conducted physically when available and online, otherwise using Skype as it was most convenient for the participants. Interviews were recorded with the permission of the participants, conducted in Arabic, translated and transcribed in English, and underwent thematic analysis using Braun and Clarke’s six-step thematic analysis.^35^ The researcher did not find a need for verbatim transcription as the purpose of transcription is to interpret and generate meaning from the data and not merely transfer audio records into words. It was effortless and time-saving to transcribe interviews in English rather than following the traditional steps of verbatim, then translation, then back-translation.^36^ Relevant literature was reviewed at each phase to support and supplement the phases’ emerging findings. The reviewed literature in three phases described a context that is fragile, conflict affected or impacted by war or crisis; examined either the delivery of healthcare by CHWs in the community or explored CHW programmes’ strengthening factors; reported a specific outcome connected to CHW programmes’ performance or community-based healthcare outcomes; and was available in English.

The research explored the topic in phase one by first reviewing relevant literature and second interviewing CHW team leaders and programme managers. The findings helped to frame the initial understanding of critical factors related to CHWs’ functionality. The initial framework was further refined and supported in phase two, where relevant literature was reviewed (third) and three NGOs were interviewed (fourth). This phase, jointly with phase one, offered a more in-depth understanding of enablers and obstacles affecting CHW programme optimisation from implementation views. Finally, building on the findings of the preceding phases, the researcher needed to (fifth) conduct a further literature review and consult two main stakeholders, governing stakeholders (sixth) and CHWs (seventh). The need to survey CHWs in the last phase emerged because of the second phase’s results which highlighted CHWs’ perception of themselves as critical as other stakeholders’ perception. In addition, the survey served as a round of verification and critique of the findings derived from previous phases. The survey fits perfectly to collect data from a large number of CHWs in a relatively short time.^37^ As for the KIIs, they were critical in helping us understand policymakers’ and governing stakeholders’ perspectives. Those stakeholders are the ones who could speak to strategic questions such as integration, funding and representation, and provide historical background on CHW programmes in Syria. The following diagram explains the sequence of the data collection process and the tools used throughout the three phases toward the final framework.

### Phase one

A review of relevant literature from low/middle-income countries and humanitarian contexts was conducted to capture evidence on what is currently known about CHW performance and optimisation within humanitarian contexts. Findings from the literature were then used to develop an interview topic guide to facilitate the first round of primary data collection.^38^

Seven NGOs with CHW programmes operating in Northwest Syria between 2013 and 2018 were invited to participate in SSIs, which were used to provide an in-depth understanding of the situation.^39^ Considering time restrictions and the exploratory nature of this phase, the researcher approached all the NGOs with CHW programme in his network. Two NGOs out of seven accepted to participate. Reasons for not participating included concerns over data use, a busy schedule or a lack of interest. However, the relatively low number of NGOs included was not a significant concern as they served the purpose of initial exploration of the topic. Additionally, the findings of this stage were further checked and confirmed in the following phases.

### Phase two

We started with reviewing up-to-date literature, as it was necessary to use recent evidence to prepare for the interviews. Then, participants were selected in consultation with experts in the field, including the CHWs’ coordination team. Considering sustainability and coverage as selection criteria, the top three CHW programmes were included. Adapting the said selection criteria was essential to allow a proper understanding of relatively stable programmes. Findings from phase two supplemented and refined the initial framework, forming the base for the third data collection phase.

### Phase three

This phase aimed to build upon the refined framework to produce a final framework for CHW optimisation on a national (Northwest Syria) scale. Accordingly, two exercises were conducted in parallel: KIIs with governing stakeholders and a survey with CHWs.

For the interviews, a stakeholder mapping was conducted to identify potential candidates and the participant list was identified in consultation with the CHWs’ coordination team. As a result, KIIs included participants from different entities that represent governing stakeholders of the current health system, as demonstrated in table 1.

**Table 1:** Interview participants per data collection phase

Category	Phase	# of interviews	Interview method
NGOs	One	6 SSIs	Online
NGOs	Two	3 SSIs	2 physical/1 online
Donors	Three	1 KII	Physical
Governing stakeholders (Health Cluster-Health Information System/Risk Communication and Community Engagement/Assistance Coordination Unit/health directorate)	Three	6 KIIs	2 physical/1 email/3 online

KIIs, key informant interviews; NGOs, non-governmental organisations; SSIs, semistructured interviews.

This stage focused on exploring the strategic aspects of CHWs’ optimal performance in the Syrian context, as identified in phases one and two: the integration/perception and representation. The interviews were conducted physically when available and online otherwise. Interviews were transcribed and thematically analysed using the six-step framework.^35^

As for the online survey, the aim was to explore CHWs’ perceptions of themselves and their work. The survey was designed by the authors and disseminated through Risk Communication and Community Engagement (RCCE) and health directorate groups. Participants included CHWs from Northwest Syria, and the survey was anonymised to eliminate any concern among participants and to ensure complete transparent answers. Survey responses were analysed statistically using pivot tables and visualised through graphs to identify and understand trends and correlations between various variables affecting CHWs’ perspectives.

### Patient and public involvement

It was not appropriate or possible to involve patients or the public in our research design, conduct, reporting or dissemination plans.

## Findings

Sixteen interviews of nine SSIs and seven KIIs were conducted with participants representing NGOs, governance stakeholders and donor groups across the three research phases (see table 1). Two hundred eighty-eight surveys were conducted with CHWs, of which 85.2% were female participants. While there is no accurate number of CHWs in the Northwest, based on the last Health Resources and Services Availability Monitoring System report issued on March 2022, there are 843 CHWs in Northwest Syria.^40^ The sample, as such, is representative, considering a margin error of 5% and a confidence level of 95%.^41^

### Phase one

**Figure 2:**
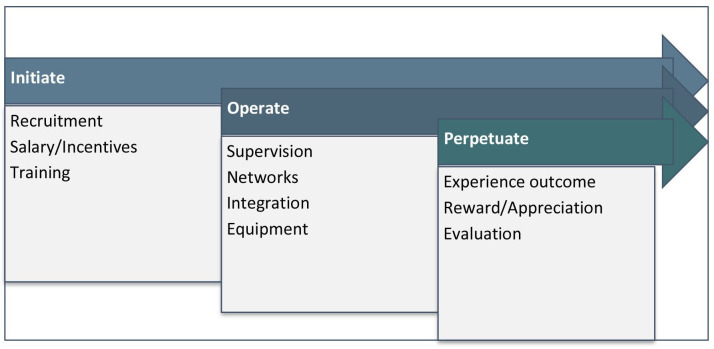
Optimisation factors derived from phase one.

Six SSIs were conducted with male CHW managers and team leaders. Findings identified 10 factors necessary for retaining effective and motivated CHWs within NGOs in humanitarian conflict settings. The factors were categorised under three main themes corresponding to an operational time frame, collectively representing optimised CHWs (figure 2).

The ‘initiate’ set of factors is the primary consideration needed to start the programme and includes essential programmatic and human resources elements like recruitment strategies, training and financial incentives. The ‘operate’ set of factors is needed for CHWs to be functional and practical throughout programme implementation. The factors include supportive supervision, active and functional networks with other service providers to support CHW referrals and linkages to non-health sectors, and integration within the overall health system to enable community engagement and trust building. Lastly, ‘perpetuate’ factors are required to maintain the CHWs’ motivation to endure the prolonged crisis. It includes reward and appreciation, witnessing their impact on the served communities and scope for professional and personal development.

### Phase two

In phase two, factors identified in phase one were revisited and expanded to include the perspectives of additional implementers. Three SSIs were conducted with representatives from Northwest’s largest and oldest CHW programmes. The findings highlight the interconnecting nature of the three themes identified in phase one. For example, adopting a recruitment criterion that includes a selection from the same community is more likely to increase community acceptance. In addition, considering communication skills as a core selection criterion will contribute to better integration with the community and the formal health cadres.

The factors identified in phases one and two were then classified under institutional and strategic components. The institutional factors include aspects concerning human resource elements which cover contracting, recruitment criteria, salary, capacity building, career path and rewarding strategies, in addition to programme aspects which include training, supervision, establishing networks with various service providers and securing supplies for community events and daily activities.

**Figure 3:**
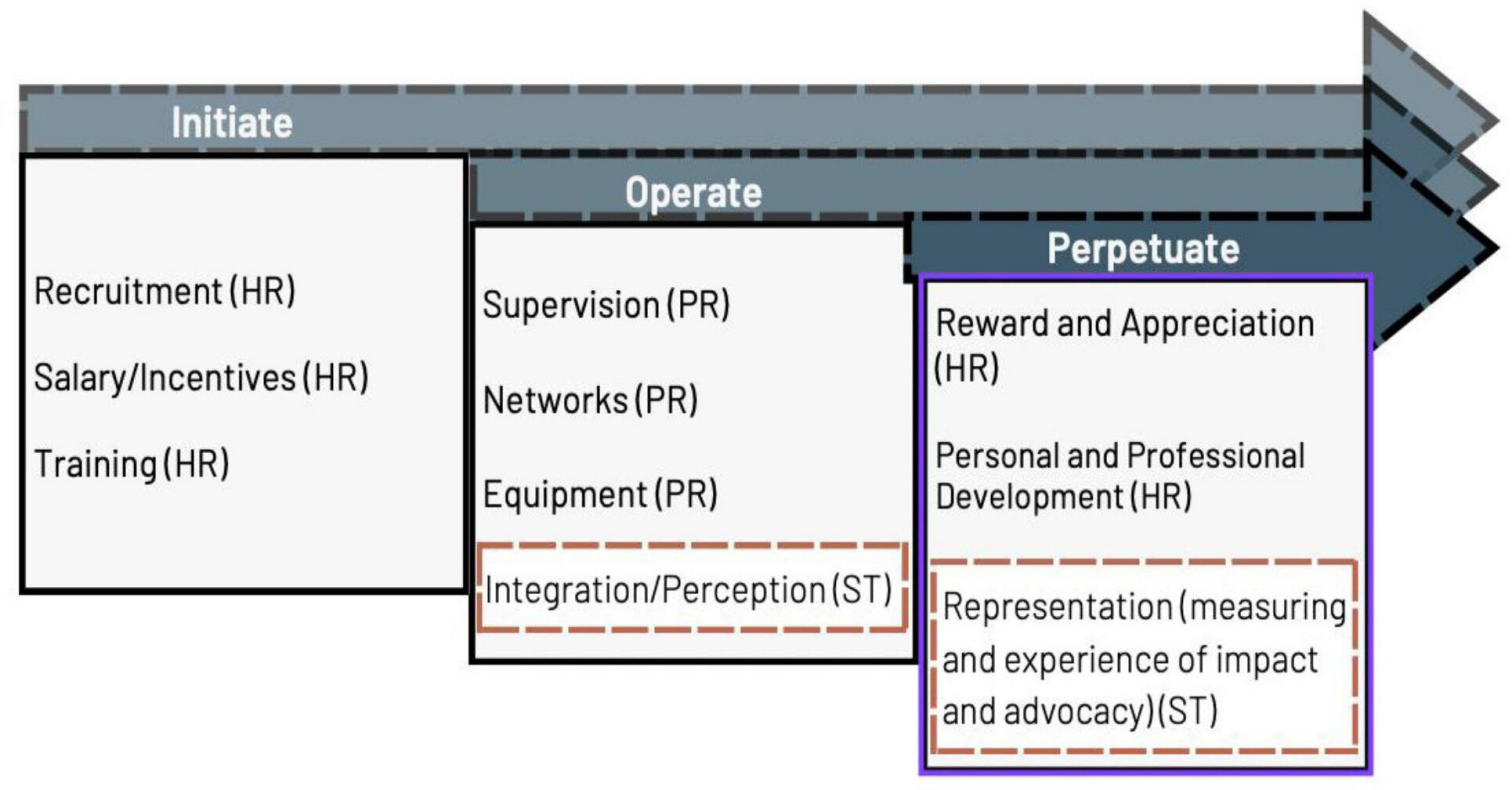
Optimisation factors derived from phases one and two. Factors in the orange boxes speak more to governing stakeholders, whereas the rest are more with implementing NGOs. HR, human resource factors; NGOs, non-governmental organisations; PR, programme factors; ST, strategic factors.

Strategic factors are concerned with broad factors relevant to decision-makers and directly impacting the extent to which CHWs contribute to strengthening health systems. These include integration and its critical determinants (perception) and impact measurement to support both CHWs (in witnessing the value of their work) and decision-makers (to build their advocacy on evidence base) (figure 3). The nine interviews conducted in phases one and two covered the institutional aspects. However, further exploring strategic components was deemed necessary, addressed in phase three.

### Phase three

Seven KIIs with decision-makers, including donors and governing stakeholders, supported the final framework’s development by exploring integration/perception and representation factors of CHWs. Additionally, a survey with 288 CHWs explored the perception factor and contributed to the final framework. Table 2 provides the demographics of survey respondents.

**Table 2:** Surveys participants’ demographic details

Demographic factor	Academic qualification				Years of CHW experience			
Categories	High school	Two-year diploma	University degree	Other	1–3	3	3–5	>5
Percentage	28 (N=80)	31 (N=91)	38 (N=109)	3 (N=8)	31 (N=90)	14 (N=40)	33 (N=95)	22 (N=63)

Demographic factor	Sex		Age			
Categories	Male	Female	19–25	26–35	36–45	>45
Percentage	15 (N=43)	85 (N=245)	19 (N=54)	62 (N=180)	17 (N=48)	2 (N=6)

CHW, community health worker.

Findings from the survey (table 3) highlight that many CHWs perceive their work as necessary and an essential component of the health system. However, when concerning how the health system or communities view their work, the CHWs did not perceive this as strongly. For example, when CHWs were asked if they recognised themselves as part of the health system, 88% strongly agreed. However, when asked if the community believed so, only 56% answered so. Eighty-nine per cent of CHWs also reported a strong belief in the value of their work, while only 50% of them are confident that the community holds the same belief.

**Table 3:** Survey results of CHWs’ perception of integration and value recognition

CHWs’ perception area	Strongly agree	Agree	Somehow agree	Disagree	Strongly disagree
Consideration as part of the formal health system					
CHWs	88% (N=253)	4.0% (N=12)	3.0% (N=9)	2.0% (N=5)	3.0% (N=9)
Local community	56% (N=161)	21.9% (N=63)	16.7% (N=48)	3.2% (N=9)	2.2% (N=7)
Specialised providers	54% (N=156)	25.1% (N=72)	11.2% (N=32)	4.5% (N=13)	5.2% (N=15)
Value recognition					
CHW	89% (N=256)	6.9% (N=20)	1.0% (N=3)	1.4% (N=4)	1.7% (N=5)
Local community	50% (N=144)	30.2% (N=87)	15.0% (N=43)	3.1% (N=9)	1.7% (N=5)
Specialised providers	58% (N=167)	21.0% (N=60)	12.5% (N=36)	5.0% (N=14)	3.5% (N=11)
Other departments	63% (N=181)	22.6% (N=65)	10.1% (N=30)	3.3% (N=9)	1.0% (N=3)
CHW managers	80% (N=230)	12.5% (N=36)	5.6% (N=16)	1.6% (N=5)	0.3% (N=1)

CHWs, community health workers.

### Synthesising findings: the optimisation framework components

Findings from phases one, two and three were synthesised to construct an overall optimisation framework. This framework comprises four core components: human resources and programme (under institutionalisation), integration and representation (under strategic). The four components must be in place to reach the optimal scalable programmes. As described in the framework, any missing component will likely hinder CHWs’ optimisation.

Adequate implementation time is a must to catalyse CHWs’ optimisation. The optimisation in this paper covers small (one NGO) and large (subnational or national) scales. When addressing the latter, more focus on political buy-in is needed as optimal NGO CHW programmes need to be replicated in other NGOs and governed appropriately. Buy-in is challenged in humanitarian conflict settings where governing structures are weakened. Nevertheless, considering that the health services are mainly delivered by international agencies collaborating with local actors, it is possible to produce successful programmes where there is will. As the KII donor highlighted, “Our biggest challenge is not funding; although it is a bottleneck, our core problem is our perception and willingness to invest when we see no evidence.”

Once CHW programmes achieve the optimal state within a given NGO, it will create an environment where evidence on what works and what does not can be generated. Such evidence will support advocacy efforts and perception change, increasing buy-in and attracting more funds.

### Institutional factors (NGOs)

#### Human resource factors

Contracting, clear recruitment criteria and job descriptions provide a sense of identity to CHWs and facilitate their integration into the more comprehensive health human resources. Financial compensation, which is sustainable and adequate, is critical for CHWs in humanitarian settings, particularly protracted ones. Capacity building was the key priority, with 41% of CHW respondents reporting it as the most important factor for motivation and retention. Appreciation strategies were identified as critical for CHWs in the long run as they allow CHWs to endure various daily operational challenges in a conflict setting. Finally, regular evaluation linked to promotion opportunities was reported as fundamental to minimising attrition.

#### Programme factors

Programme factors are those related mainly to programme managers accountable for supervising, equipping, introducing and linking their programmes to other service providers. Supervision was seen as a representation of appreciation and the leading resource for supporting CHWs in the field. It was frequently described as fundamental to retaining effective CHW teams. Programme managers must also ensure proper linkages between CHWs and non-health service providers such as education, protection, livelihood, and water, sanitation and hygiene. As described by an NGO participant, “Our community needs not only health messages. It did not make sense for us to teach families about lice prevention when we know they do not have access to lice shampoo.” Finally, CHWs must have all the supplies to facilitate their community outreach activities.

### Strategic factors (national and subnational)

#### Integration/perception

Integration is critical for CHWs’ effectiveness; without it, CHWs’ contribution was limited. Integration was characterised as active two-way referral pathways between the community and health system, meaningful utilisation of the CHW data by health information systems, and formal training and supervision by the governing entities, leading to recognition as part of the health system. Perception (of all the involved stakeholders, including CHWs, governing bodies, donors, NGO workers and formal health cadres) was presented as an integral determinant for integration. Poor perception can hinder integration efforts even when funding is available.^42^

#### Representation

Impact measurement and experiencing outcomes: representation is the ability to measure and present impact to the public, including the CHWs. Representation was an underpinning cause for various challenges hindering CHW optimisation, such as the perception of key stakeholders. One KII explained, “The investment in CHW programmes nationally is not a simple decision, especially in our conflict context and restricted funding opportunities. Investment in CHWs is more challenged when we have no evidence for impact. Our current perception of CHWs, even when we know that it has been affected by the inappropriate implementation of the programme, is our main challenge.” The importance of measuring the impact and representing it to the broader community was also reported by an NGO participant who elaborated, “Presenting CHWs impact and appreciating their contribution is fundamental. We need them to recognise that their role is critical, and it is saving lives.” Another NGO participant elaborated, “Appropriate tools have to be tailored to help us not only report on short-term output figures but, more importantly, measure the impact.”

## Discussion

The optimisation framework highlights the need for coordination between programme implementers and national and subnational leadership. To achieve optimal CHW programmes, it is crucial to have adequately trained and equipped human resources, sufficient implementation time, integration with the broader health system and relevant actors, setting strategies to ensure the impact is visible and the CHWs are credited with any success. Failure of one or more of these critical constructs can result in ineffective, limited and unknown programmes. The requirements for optimising CHW programmes in protracted conflict settings are like other fragile settings. However, conflict-associated factors put more stress on the importance of strategic integration and impact measurement. In Northwest Syria, a mix of emergency and development conditions is presented. Accordingly, flexibility in funding policies is critical. In addition, considering that CHWs in Syria are mainly assigned to health education and awareness raising, their contribution is perceived as limited.

Contrary to other settings where the community appreciates the curative role CHWs have,^22^ community appreciation and a sense of achievement underpinned the need to expand the social role of CHWs in the Syrian context. Social roles mean linking communities with various services available and supporting families in accessing these services. Other works detailing improving or optimising CHW performance can further support these findings.

**Figure 4:**
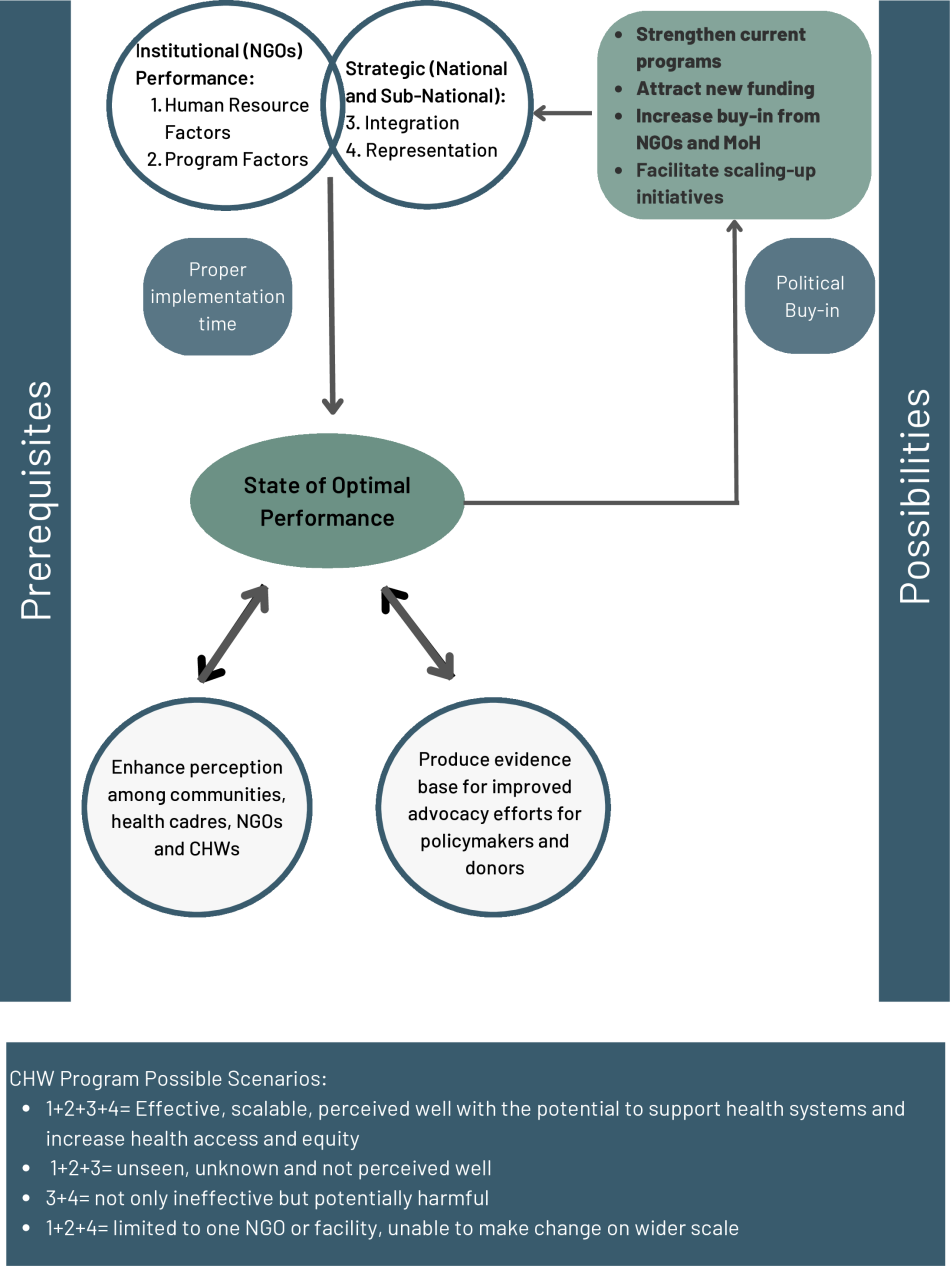
Optimisation Framework for CHWs in humanitarian conflict settings.

In 2019, the Human Resource for Health 2030 programme’s CHW policy implementation enabler framework identified three stages: build, manage and optimise, under which various factors were stated.^43^ While not specifically for humanitarian contexts, this framework provides 15 items that describe key considerations across these stages, aligned with the our CHW optimisation framework (figure 4). Notably, this work includes aspects of CHW selection and community engagement which can be further discussed in a dedicated study.

Others have also attempted to document CHW performance optimisation in humanitarian contexts.^23^ Organisational theories, such as organisational commitment, organisational justice and need satisfaction identified within this, can explain our findings, specifically around CHW perception and human resource factors. For example, the need for CHWs to commit to the NGO (organisational commitment) can be influenced by one’s respect and aligned ethos; however, role insecurity and lack of sustainability may reduce commitment. It may also be necessary for CHWs to perceive fairness within their work and feel they have autonomy in decision-making and their role. Such factors can explain key constructs in our framework, further unpacking critical concepts within the CHW humanitarian field.

The US AID tool for Assessment and Improvement of CHWs^44^ complemented the WHO 15 recommendations guidelines.^45^ It includes 10 assessment aspects of CHW programmes. However, the tool, similar to the WHO guideline, did not address non-government settings where no Ministry of Health exists. This research presented additional factors for CHWs’ functionality, such as perception as a determinant for integration. Furthermore, the need to witness the impact was also found as a necessity for CHW retention, leading to the fourth central theme in the final theory, representation. This aligns with the WHO’s call for continued collection and evaluation of data regarding CHW performance and impacts.^46^

In the Syrian context, the health system heavily depends on external humanitarian funding, which has these actors deciding on what gets implemented, where and by whom. Nevertheless, this research showed that local actors mainly implement CHW programmes. Accordingly, implementing partners and local actors should be accountable for progress, including monitoring and assessment. While donors can steer and guide actions toward specified objectives, the ultimate responsibility should be within local hands. The emerging locally led health response through local NGOs and bottom-up health governing bodies is a promising platform to develop the work of CHWs further with better integration with the local health systems.^47 48^

The indicators currently measuring CHWs’ outcomes in Syria are mainly developed to serve donors’ reporting and cannot present CHWs’ work comprehensively. For example, current data show that CHWs report mainly about the number of households visited, the number of beneficiaries who attended awareness sessions and the number of referrals. Although such indicators serve as an index for coverage and workload, they fall short of adequately describing the final result of these activities. This, therefore, has repercussions for representation within the described framework and limits CHW optimisation.

Improving monitoring within Syria needs to be considered from the design stage of CHW programmes and supported with budgeted resources and relevant capacity building. Indicators such as the percentage of women completing antenatal care/postnatal care visits, exclusively breast feeding, coverage of vaccines or under-5 mortality rates are highly needed to be applied, in addition to indicators that will capture CHW integration and perceptions. As the Johannesburg, South Africa conference for strengthening CHW institutionalisation stated, “all community health programmes require detailed, prospectively designed implementation research to fill evidence gaps, inform policy and enable course correction in real time.”^49^ The role of CHWs can be hindered by unrealistic expectations, lack of a clear focus and poor documentation. Therefore, further research considering rigorous suitable study design, documentation of CHW activities, and carefully defined indicators and target populations are needed.^50^

Further work in this area should be done to strengthen CHW programming within humanitarian contexts. Research that unpacks each component to provide detailed steps and recommendations for strengthening implementation can be done. Given the limited data on CHW programmes in Syria, implementers are encouraged to disseminate their work and prioritise implementation research that can improve our understanding of CHW programmes in these contexts.

### Limitations

A genuine effort was made to include female leaders of CHW programmes or from the governance stakeholders, but the interviewees included only males. This is due to a gap in this field. However, such a gap was not found within the CHW level, as 85% of survey respondents were females. The paper presented the optimisation framework with its four components, but it was not feasible to discuss in depth each one of these components. Follow-up work is needed to understand each factor and what practices best fit the Syrian context. The interpretive approach suggests that the research question can be defined and answered in many ways based on individual and social perceptions.^51^ The researcher acknowledged that his position, experience and understanding of the context and the topic shaped the research findings and recommendations. Five out of 16 interviewees were the researcher’s colleagues, which helped make the interviews less formal but more informative since the stress factor was eliminated. On the other hand, it might have increased the likelihood of researcher expectations influencing the findings. The researcher made a genuine effort to minimise this influence during the interviews by repeating questions and asking for supporting details to ensure the interviewees’ answers were genuine and not only driven by the natural tendency to agree.

## Conclusion

Although the concept of CHWs was relatively new to the Syrian health system as NGOs introduced it after the start of the conflict, CHWs played a key role in the health response through vital community outreach activities that complemented the work of fixed health facilities. More than 10 years of health response in Syria allowed health providers and NGOs to explore various mechanisms to optimise the work of CHWs. Learning from this case study is essential to add to the relatively limited literature on CHW optimisation in conflict settings.

The final framework has presented a formula for optimising CHW programmes, which includes four interconnected components: human resource, programme factors, integration and representation. The optimal state in this analysis is the outcome of these factors’ interaction. While CHW programmes implemented in conflict settings present incredibly challenging conditions, donors and implementers must prioritise how best to support CHWs and consider their needs throughout design and implementation. In such settings, CHWs can play a vital role in strengthening community outreach components of health systems. If adequately supported, it can improve the efficiency of humanitarian response and pave the way for a community-led health system in early recovery and post-conflict.

10.1136/bmjgh-2023-011837.supp1 Supplementary data

## Data Availability

No data are available.
