# Correction: A framework for community health worker optimisation in conflict settings: prerequisites and possibilities from Northwest Syria

**DOI:** 10.1136/bmjgh-2023-011837corr1

**Published:** 2023-08-17

Habboush A, Ekzayez A, Gilmore B. A framework for community health worker optimisation in conflict settings: prerequisites and possibilities from Northwest Syria. *BMJ Glob Health* 2023;8:e011837. doi: 10.1136/bmjgh-2023-011837

In the published version the figure 4 had has been changed to fix an error in last line.

